# Correction: Skilled birth care uptake among women from socially disadvantaged minorities in the Kambata-Tambaro Zone, Southern Ethiopia

**DOI:** 10.1371/journal.pgph.0004043

**Published:** 2024-12-04

**Authors:** Abebe Alemu, Biruk Assefa, Ritbano Ahmed, Hassen Mosa, Negesso Gebeyehu

In Table 3, the confidence interval values of some variables are incorrect. Please see the correct Table 3 here.

**Table 3:** Factors associated with skilled birthing care service among socially marginalized women, Kambata-Tembaro zone, Southern Ethiopia, 2019.

Variables	Skilled birthing care utilized		COR	aOR
	Yes	No		
**Education level of the mother**				
Can’t read and write	253(55.8)	200(44.2)	1	1
Primary& secondary	50(62.5)	30(37.5)	1.8(1.42–4.33)	1.2(2.2–5.02)
Diploma and above	4(57.1)	3(42.9)	2.4(1.79–3.03)	3.5(1.79–4.03)
**Occupation**				
House-wife	100(20.6)	385(79.4)	1	1
Employed	20(80)	5(20)	1.28(1.11–2.09)	2.49(1.50–6.5)
**Pregnancy planned**				
Yes	55(57.9)	40(42.1)	1	1
No	105 (25.3)	310(74.7)	0.86 (0.35–9.39)	0.43(0.19–8.89)
**Antenatal care visit**				
Yes	65(56.5)	50(43.5)	1	
No	115(29.1)	280(78.9)	0.321 (0.30–0.83)	0.75(0.6–8.63)
**Mothers life-style**				
Yes	118 (37.1)	200(62.9)	0.54(0.25–7.25)	0.83(0.47–9.16)
No	104(54.1)	88(45.8)	1	1
**Awareness on birthing care**				
Yes	102(56.6)	78(43.3)	1.41(1.14–2.39)	4.36(4.02–7.02)
No	95(28.8)	235(71.2)	1	1
**Number of birth**				
1–4	104(49.5)	106(50.5)	2.13(1.16–4.09)	3.23(2.03–5.65)
≥ 5	186(62)	114(38)	1	
**Respectful birthing care**				
Yes	35(70)	15(30)	2.12(1.23–8.09)	5.05(2.36–6.89)
No	33(73.3)	12(26.7)	1	1
**Social discrimination affects service uptake**				
Yes	80(40.4)	118(59.6)	0.35(0.23–0.89)	0.56(0.36–0.70)
No	150(48.1)	162(51.9)	1	1
