# Supplementary material for: Skilled birth care uptake among women from socially disadvantaged minorities in the Kambata-Tambaro Zone, Southern Ethiopia
Source: PLOS Glob Public Health. 2022 Nov 10;2(11):e0001238. doi: 10.1371/journal.pgph.0001238 (PMC10021963; doi:10.1371/journal.pgph.0001238)
Supplement: Attachment — Submitted filename: Response to Reviewers.docx [file pgph.0001238.s001.docx]

**Dear, Reviewers!**

First of all, I would like to extend my heartfelt appreciation for your scientific comments and suggestion on the manuscript submitted. Hereby, I tried to respond to your some question one by one, but I accept and amend all your valuable comments and suggestion in the manuscript.

| Reviewer #1: | |
| --- | --- |
| Q1 | - It is helpful to the reader of your paper if you can show the frequencies (percentages) by the **outcome variable**. This applies to Tables 1, 2, and 3. |
| Answer | - Outcome variable of the study is **(skilled birth care utilization)** which is mentioned in Table_2; 3^rd^ variable. The frequency and percentage of the outcome variable is mentioned there. Particularly, in Table-1 and 2, all variables were shown by frequency and percentage. - *NB: I’m okay, to have a comment if anything is missed!* |
| Q2 | - I do not think that Figure 1 is useful. |
| Answer | - Yes, I removed it, because the magnitude of skilled birth care utilization is indicated in the table_2 (which is 19%) |
| Q3 | - In table 3, the total number of the education level of the mother is 540 (253+200+50+30+4+3) > 510. Please check the frequencies. |
| Answer | - Yes, I corrected it! 🡺253+200+30+20+4+3 = 510 |
| Q4 | - Did you consider a survey design technique when you performed the statistical analysis? |
| Answer | - Yes, a community-based cross-sectional study design was used. This is indicated in the first paragraph of the method and materials part. |
| Q5 | - Did you use a standard logistic regression model or a logistic regression for a survey design? |
| Answer | - Yes, Logistic regression was used for analysis. Because logistic regression is a statistical analysis method to predict a binary outcome variable. Hereby, “ skilled birth care service utilization”, used or not used, - I think, logistic regression is a statistical analysis method that is appropriate. |
| Q6 | - On page 9, "Both bivariate and multiple variable logistic regression analyses were used to determine the association of independent variables with outcome variables." Please define the outcome variable. |
| Answer | - Yes, the outcome variable is defined, under the subtitle “Data management and analyses”, on the fourth line |
| Q7 | - Please define your primary aim. |
| Answer | - To assess the magnitude of skilled birth care uptake and associated factors among women from socially disadvantaged minorities |
| Q8 | - Was the sample size determination performed for analysis in Table 3? Please clarify. Do you have a reference for the sample size determination? |
| Answer | - Sample size was determined by the “Single population formula” and there was a “typing error” in table_3, regarding sample size, it is corrected and the sample size was referenced (under the subtitle ‘sample size determination’, online,2) |
| Reviewer#2 | |
| Q1 | - Overall manuscript preparation style - Methodology, ethical review, findings, and discussion sections - Grammar - Referencing style etc. |
| Answer | - Yes, all given comments and suggestions were very constructive. - I tried to revise the “whole manuscript” as per your comments and suggestion as much as possible. - All changes or revisions made will be submitted with ***“manuscript with track change”*** |
